# Metabolic and clinical effect of alpha-lipoic acid administration in schizophrenic subjects stabilized with atypical antipsychotics: A 12-week, open-label, uncontrolled study

**DOI:** 10.1016/j.crphar.2022.100116

**Published:** 2022-06-28

**Authors:** Fiammetta Iannuzzo, Gianpaolo Antonio Basile, Domenica Campolo, Giovanni Genovese, Gianluca Pandolfo, Loretta Giunta, Domenica Ruggeri, Antonino Di Benedetto, Antonio Bruno

**Affiliations:** a Department of Biomedical and Dental Sciences and Morphofunctional Imaging, University of Messina, Via Consolare Valeria 1, Contesse, Messina, 98125, Italy; b Psychiatry Unit, Polyclinic Hospital University of Messina, Via Consolare Valeria 1, Contesse, 98125, Messina, Italy; c Department of Internal Medicine, Polyclinic Hospital University of Messina, Via Consolare Valeria 1, Contesse, 98125, Messina, Italy

**Keywords:** Alpha lipoic acid, Metabolic syndrome, Schizophrenia, Second-generation antipsychotics

## Abstract

**Background:**

Many of the atypical antipsychotics induce metabolic side effects, limiting their use in clinical practice. Alpha-lipoic acid (ALA) was proposed as a new approach in schizophrenia to improve metabolic effects of atypical antipsychotics. The aim of the study is to evaluate the effect of ALA on metabolic and clinical parameters among schizophrenic subjects.

**Methods:**

15 schizophrenic subjects, in stable atypical antipsychotic monotherapy were included in the study. ALA was administrated at the oral daily dose of 600 ​mg/d in addition to antipsychotic therapy. Metabolic, clinical, and psychopathological parameters were measured at typical antipsychotics. e initial screening, and after 12 weeks.

**Results:**

ALA produced a statistically significant reduction in QTc (*p* ​= ​*0.012*), blood glucose (*p* ​= *0.005*), AST (*p* ​= ​*0.021*), γGT (*p* ​= ​*0.035*), CPK (*p* ​= ​*0.005*) and prolactinaemia (*p* ​= ​*0.026*). In contrast, there was a significant increase in HbA1c (*p* ​= ​*0.026*). No effects on body weight and blood lipid levels (triglycerides, total cholesterol, HDL, LDL) emerged.

**Conclusions:**

ALA treatment appeared to be effective for reducing diabetes risk, liver functionality parameters, hyperprolactinaemia and QTC interval. ALA appears to be safe as adjunctive components in schizophrenia.

## Highlights

• Atypical antipsychotics induce the frequent occurrence of metabolic side effects.
• Prevalence rates of Metabolic Syndrome in patients with schizophrenia are high.
• No specific therapies for metabolic effects of SGAs in schizophrenia are still available.
• ALA was suggested as a potential therapeutic agent in various chronic diseases.
• ALA appeared to be effective to improve metabolic values in schizophrenia.

## 1 Introduction

Despite their clinical efficacy, many second generation antipsychotics can induce metabolic side effects such as dyslipidaemia, hyperglycaemia, weight gain, and/or Metabolic Syndrome (MetS), thereby limiting their use in clinical practice (Haddad and Wieck, 2004; Malhotra et al., 2013). However, atypical antipsychotics are not a homogeneous class of drugs and are associated with different side effect profiles. For example, olanzapine and clozapine are associated with the highest risk of metabolic complications, whereas quetiapine, risperidone, asenapine, and amisulpride can cause moderate metabolic alterations (Carli et al., 2021). Although the exact relationship between atypical antipsychotics and metabolic alterations is still uncertain (Misawa et al., 2011; Reynolds and Kirk, 2010), studies suggest that weight gain and dyslipidaemias rather than hypertension, are the side effects more associated with this drug class (Hirsch et al., 2017). The pharmacodynamic effects of SGAs on histaminergic (H1), serotonergic (5-HT1A and 5-HT2C), and dopaminergic (D2) receptors can directly cause increased appetite and high caloric intake (Kroeze et al., 2003; Sezlev-Bilecen et al., 2016), while antipsychotic-associated dyslipidaemia is thought to derive from peripheral and hypothalamic pathophysiological mechanisms. Another mechanism through which this side effect is induced is that SGAs seem to increase lipogenesis through the up-regulation of sterol regulatory element-binding transcription factors (SREBF) involved in lipid biosynthesis (Yang et al., 2007).

Management and reduction of metabolic side effects remain a major concern when considering atypical antipsychotics for the treatment of schizophrenia. While there is a general consensus regarding routine monitoring of metabolic syndrome indices in patients receiving atypical antipsychotics, adherence to the guidelines is not common (Burghardt and Ellingrod, 2013) and no specific therapeutic interventions are available, meaning that metabolic complications in schizophrenic patients are treated as in the general population. According to a recent metanalysis, metformin, which increases glucose transporter 4 (GLUT4) mRNA expression, appears to be effective in treating the antipsychotic-related weight gain in patients with schizophrenia or schizoaffective disorder, with more efficacy in first episode compared to chronic patients (de Silva et al., 2016). Conversely, evidence regarding the efficacy of statins, which inhibit the hydroxymethylglutaryl Co-A reductase, to treat atypical antipsychotics-induced dyslipidaemia is still sparse (Ojala et al., 2008; Tse et al., 2014; Vincenzi et al., 2014). Considering that these therapeutic strategies often present additional side effects (Alsheikh-Ali and Karas, 2009; Flory and Lipska, 2019; Joy, 2009; Pfeiffer and Klein, 2014), there is the need to investigate alternative treatment strategies. Within this framework, the nutraceutical approach might be a promising strategy in the management of diabetes mellitus, dyslipidaemia, MetS, and the complications associated with them, to significantly modulate the biochemical and clinical end-points of interest (Premi and Bansal, 2021; Sarris et al., 2022).

Alpha lipoid acid (ALA) is a natural substance commonly found in dietary components such as vegetables and meats, which is an essential cofactor of mitochondrial respiratory enzymes and is crucial for normal functioning of oxidative metabolism (Shay et al., 2009). ALA plays several biochemical roles including antioxidant function, metal chelation, and modulation of signal-transduction pathways, like insulin and nuclear factor kappa B (NFkB) (Golbidi et al., 2011). Furthermore, it also seems to improve endothelial dysfunction through a protective activity against atherosclerosis development and progression (Wray et al., 2012; Ying et al., 2010; Zhang et al., 2008), altogether justifying the use of ALA as a potential therapeutic agent in various chronic diseases such as MetS (Packer et al., 2001), Alzheimer's disease (Moreira et al., 2007), cognitive dysfunction, and some forms of cancer (AL Abdan, 2012).

The therapeutic use of ALA in schizophrenia has recently been investigated in human populations. A case series by Kim et al. (2008) explored the efficacy of ALA as a novel agent to treat antipsychotic-induced obesity, at a dose of 1200 ​mg/d (range between 600 and 1800 ​mg/d); reporting the key effect to be a reduction in body weight and BMI after a 12-week treatment. In a pilot open-label trial, Sanders et al. (2017) administrated 100 ​mg/d of ALA as a general adjuvant to antipsychotics therapy, with no significant improvement in BMI, abdominal circumference, blood count, or liver enzymes. Finally, Vidović et al. (2017) investigated the effects of 500 ​mg/d of ALA on plasma adiponectin levels, fasting glucose, and aspartate aminotransferase activity, with no significant effect on the metabolic parameters.

Based on this background, ALA may be a potentially interesting therapeutic agent to improve the metabolic effects of atypical antipsychotics. The purpose of this study was to assess: (1) the efficacy of ALA on metabolic factors and (2) its safety and potential therapeutic effects in a sample of schizophrenic patients in stable therapy with atypical antipsychotics.

## 2 Materials and methods

### 2.1 Study design

This was a 12-week, open-label, uncontrolled study, which aimed to evaluate the efficacy of adjunctive ALA in a sample of schizophrenia patients receiving atypical antipsychotic therapy. ALA was administrated in capsules at a fixed oral daily dose of 600 ​mg for the entire duration of the study in addition to the atypical antipsychotic therapy. The maximum dose of 600 ​mg per day was established according to Kim et al. (2008) to avoid severe side effects.

### 2.2 Subjects

The accidental sampling method was used to recruit the participants. Patients who met DSM-5 criteria for schizophrenia, aged between 18 and 60 years old in stable atypical antipsychotic monotherapy (clozapine, olanzapine, quetiapine, or risperidone) for least 3 months (Carli et al., 2021) were consecutively selected among the outpatients of the Psychiatry Unit of the University Hospital of Messina, Italy. The drug dose remained unchanged throughout the investigation and no additional medications (antidepressant/anticonvulsant/anxiolytic/anti-inflammatory) were added to their drug regimen during the study. At enrollment, all subjects received dietary advice to maintain their alimentary habits unchanged, to reduce the possible influence of dietary restrictions on metabolic values during the study period. Exclusion criteria included treatment with more than one atypical antipsychotic, current treatment with insulin/oral hypoglycaemic/lipid-lowering agents, significant concomitant medical pathologies, organic brain disorders, history of alcohol or substance dependence (excluding nicotine), dementia, mental retardation, and pregnancy/breastfeeding.

All the patients provided written informed consent after a full explanation of the experimental design, according to the Declaration of Helsinki. Patients were recruited from June 2021, and the follow-up was completed in December 2021.

### 2.3 Assessment

Patients attended two visits: initial screening (day 0), and final visit (week 12). Standard laboratory methods were used to determine, as primary outcome measures, fasting levels of glucose, glycated haemoglobin (Hb1c), total cholesterol, high-density lipoprotein (HDL) cholesterol, triglycerides, and as secondary outcome measures, alanine aminotransferase (ALT), aspartate aminotransferase (AST), gamma-glutamyl transferase (γGT), creatine phosphokinase (CPK), uric acid, creatinine, azotaemia, and prolactinaemia. Moreover, a physical examination was performed to measure systolic and diastolic blood pressure, heart rate, body weight, and body mass index (BMI). Electrocardiography (ECG) tracing was performed to measure QT interval and QTc. Finally, psychopathologic symptoms were assessed using the Positive and Negative Schizophrenic Symptoms Scale (PANSS) (Kay et al., 1987), which was administered by two senior psychiatrists with at least 5 years of clinical experience and previously trained regarding rating scales. The same person conducted the clinical interviews and the psychometric tests for each patient. All physical information, laboratory measurements, and data for clinical assessments were obtained at baseline and the end of the study (approximately 12 weeks apart). Adverse effects, either observed or spontaneously reported, were recorded and classified for onset, duration, severity, and outcome. To monitor the adherence to the study protocol, weekly telephone calls during the study period and a pill count on the final visit were carried out.

### 2.4 Statistical analysis

Prior to the start of the study, a power and sample size estimation was conducted (G∗Power 3.1.9.2.), under the assumption of an effect size of 0.8, a significant level of 0.05 with a power of 0.80, a minimal sample size of 12 was determined. Considering dropouts to normally be around 20%, a final sample size of 15 was selected for the study.

Due to the small sample size, no randomization/allocation in subgroups was performed, and the analysis was carried out by nonparametric tests. Given the absence of dropouts, no last observation carried forward analysis was performed. Continuous data were expressed as mean (±S.D.), and the within-group differences between baseline and final tests were analysed by the Wilcoxon rank-sum test for dependent samples. As for the magnitude of the treatment effect, effect size was calculated based on Cohen’s d statistic and values lower than 0.50, ranging from 0.50 to 0.79, and 0.80 or greater, were considered small, moderate, and large, respectively (Cohen J., 1988). Results were considered significant for p values ​< ​0.05. The statistical analysis was performed using the Statistical Package for the Social Sciences (SPSS) 25.0 software (SPSS Inc, Chicago, IL, USA).

## 3 Results

**Fig. 1:**
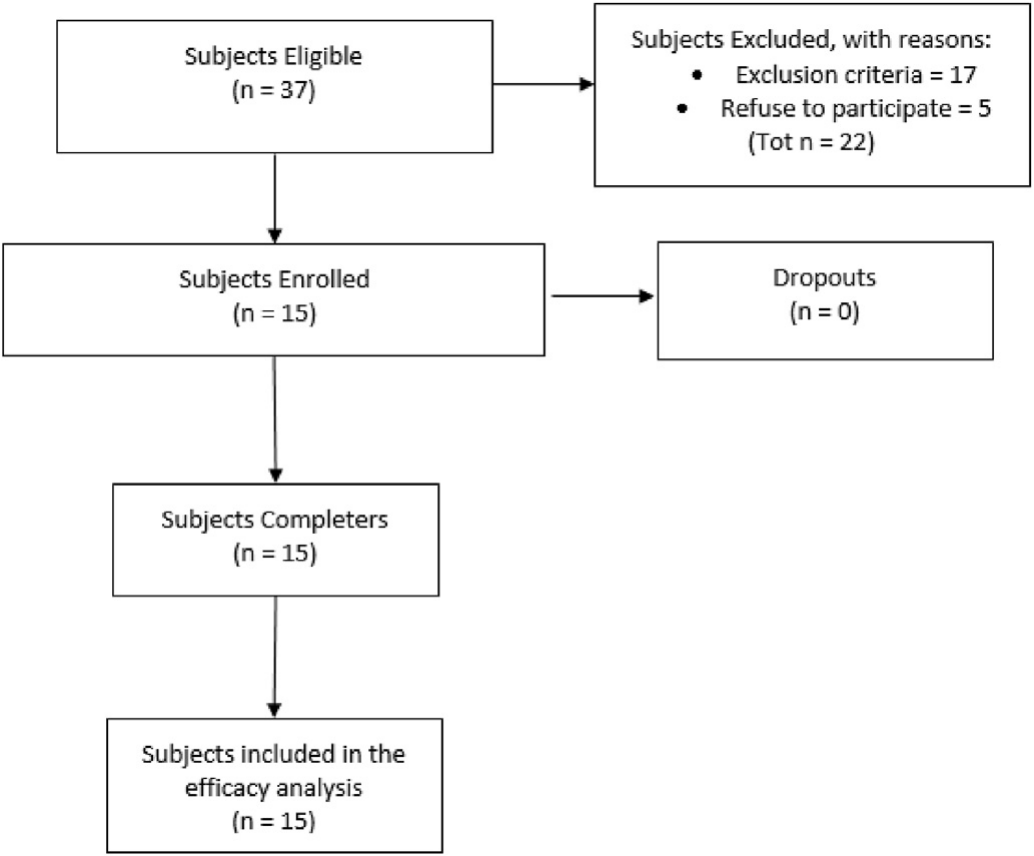
Flow Diagram of patient recruitment.

Fig. 1 summarizes the flow chart of patient recruitment. From a pool of 37 eligible patients, 17 were excluded according to the exclusion criteria and 5 because of refusing to participate in the study. Fifteen subjects (11 Male and 4 Females) met the inclusion criteria and thus were included in the study. All the enrolled subjects completed the research protocol (100% completion rate) and were considered for the efficacy analysis.

Baseline characteristics, duration of illness, drug type and dose range in final sample are detailed in Table 1.

**Table 1:** Demographic and clinical characteristics of the sample.

Patients enrolled (completers)	15/15
Sex (M/F)	11/4
Age, mean ​± ​SD, years	44.60 ​± ​11.16
Educational level, mean ​± ​SD, years	10 ​± ​2.58
Duration of illness, mean ​± ​SD, years	23.80 ​± ​10.37
Atypical Antipsychotics (Range dose – mg/d)	**N**
Clozapine (150–300 ​mg/d)	3
Olanzapine (10–20 ​mg/d)	5
Quetiapine (400–800 ​mg/d)	4
Risperidone (2–4 ​mg/d)	3

Table 2 shows values of the clinical and metabolic parameters assessed at baseline and at the end of the study in addition to the effect size for the sample group. At week 12, statistical analysis showed that ALA administration produced a statistically significant reduction in QTc (QTc T0 vs T1 (mean ​± ​SD) ​= ​422.8 ​± ​17.11 vs 408.8 ​± ​21.74, *p* ​= ​*0.012*), blood glucose (Glucose T0 vs T1 (mean ​± ​SD) ​= ​105.4 ​± ​14.05 vs 88.8 ​± ​12.56, *p* ​= ​*0.005*), AST (AST T0 vs T1 (mean ​± ​SD) ​= ​23.2 ​± ​7.16 vs 17.8 ​± ​3.36, *p* ​= ​*0.021*), γGT (γGT T0 vs T1 (mean ​± ​SD) ​= ​18.2 ​± ​7.06 vs 15.6 ​± ​6.13, *p* ​= ​*0.035*), CPK (CPK T0 vs T1 (mean ​± ​SD) ​= ​203.4 ​± ​177.13 vs 84.2 ​± ​50.35, *p* ​= ​*0.005*) and prolactinaemia (Prolactinaemia T0 vs T1 (mean ​± ​SD) ​= ​389.76 ​± ​405.65 vs 190.16 ​± ​196.75, *p* ​= ​*0.026*). In contrast, there was a significant increase in HbA1c (HbA1c T0 vs T1 (mean ​± ​SD) ​= ​5.2 ​± ​0.53 vs 5.34 ​± ​0.62, *p* ​= ​*0.026*) and azotaemia values (Azotaemia T0 vs T1 (mean ​± ​SD) ​= ​25.6 ​± ​4.14 vs 30.8 ​± ​4.91, *p* ​= ​*0.021*), both of which remained within the normal range. No significant differences in other clinical (body weight, BMI, and blood pressure) and metabolic parameters (triglycerides, total cholesterol, HDL, and LDL) were observed. Effect sizes were large in Glucose (*d* ​= ​*1.26*), AST (*d* ​= ​*0.9*), CPK (*d* ​= ​*0.9*) and Azotaemia (*d* ​= ​*1.1*), moderate in QTc (*d* ​= ​*0.7*) and Prolactinaemia (*d* ​= ​*0.6*), and small (*d ​< ​0.50*) in other clinical and metabolic parameters explored.

**Table 2:** Clinical and metabolic changes and effect sizes for efficacy measures in patients receiving alpha-lipoic acid at baseline and week 12.

	Baseline (T0)		Week 12 (T1)		Wilcoxon test T0 vs T1	Cohen d
	Mean	S.D.	Mean	S.D.	p	
Body weight (kg)	95.80	19.13	96.48	17.20	.441	0
Body mass index (kg/m2)	32.18	5.99	32.84	5.63	.122	0.1
Systolic blood pressure (mmHg)	137.00	22.01	129.00	18.07	.165	0.4
Diastolic blood pressure (mmHg)	82.00	15.49	84.00	14.68	.670	0.1
QTc (msec)	422.80	17.11	408.80	21.74	.012	0.7
Triglycerides (mg/dl)	110.60	57.01	132.00	78.55	.237	0.3
Total cholesterol (mg/dl)	177.20	39.62	180.60	39.93	.798	0.1
HDL (mg/dl)	38.80	6.74	37.60	7.56	.382	0.2
LDL (mg/dl)	108.80	33.93	112.60	41.53	.441	0.1
Glucose (mg/dl)	105.40	14.05	88.80	12.56	.005	1.2
HbA1c (%)	5.20	.53	5.34	.62	.026	0.2
ALT (U/L)	20.80	12.06	21.80	9.76	.719	0.1
AST (U/L)	23.20	7.16	17.80	3.36	.021	0.9
γGT (U/L)	18.20	7.06	15.60	6.13	.035	0.4
CPK (U/L)	203.40	177.13	84.20	50.35	.005	0.9
Creatinine (mg/dl)	.86	.17	.88	.13	.458	0.1
Azotemia (mg/dl)	25.60	4.14	30.80	4.91	.021	1.1
Prolactinemia (μUl/ml)	389.76	405.65	190.16	196.75	.026	0.6

No significant changes were observed in the subscales and PANSS total score (Table 3), suggesting that psychopathological symptoms did not worsen during the trial. The administration of ALA at a dose of 600 ​mg/day was generally well-tolerated and no patient presented neither adverse effects nor undesirable change in the safety parameters. Finally, no acute extrapyramidal effects, seizures, or adverse cardiac events occurred.

**Table 3:** Psychopathological changes and Effect Sizes for efficacy measures in patients receiving alpha-lipoic acid at baseline and week 12.

	Baseline (T0)		Week 12 (T1)		Wilcoxon test T0 vs T1	Cohen’s d
	Mean	S.D.	Mean	S.D.	p	
PANSS						
Positive	12.40	3.02	13.20	5.55	.776	0.2
Negative	20.40	11.95	20.80	13.08	.776	0
Gen. Psychopathology	40.00	14.36	40.80	20.73	.798	0
Total score	72.80	28.64	74.80	34.21	.719	0.1

## 4 Discussion

Schizophrenia patients show a high incidence of MetS with prevalence rates much higher than that of the general population and healthy controls (Casey, 2005). Although schizophrenia by itself is a risk factor for MetS, strong evidence relates metabolic consequences to the long-term use of atypical antipsychotic medications.

Undoubtedly, the metabolic effects of atypical antipsychotics represent a serious medical problem that needs to be addressed with specific therapeutic approaches to improve quality of life and life expectancy of patients with mental disorders. Pharmacological treatments have been proposed to reduce antipsychotic-induced metabolic abnormalities, but standard treatments have shown limited efficacy and pose safety concerns, especially in patients with a long disease history (de Silva et al., 2016). As adjunctive treatment with ALA seems to be a promising avenue to improve quality of life, lipid metabolism, and weight reduction (Salehi et al., 2019), the current study was designed to assess the efficacy of ALA on metabolic values in a sample of schizophrenia patients stabilized with atypical antipsychotics.

The main finding of the current work is that patients treated for 12 weeks with ALA at 600 ​mg/d experienced highly significant improvement of blood glycaemia, while glycated haemoglobin, although showing a tendency to worsen, is maintained within normal values. This result indicates the effect of ALA in reducing the risk of MetS since hyperglycaemia is the most ascertained mechanism in the pathogenesis of diabetes. In addition, this result is consistent with the previous findings showing a dose-dependent decline in glycaemic parameters in diabetic patients treated with ALA at different daily doses (Porasuphatana S et al., 2012).

No direct effects on body weight and blood lipid levels (triglycerides, total cholesterol, HDL, LDL) were observed in our sample. This result is in accordance with other previous studies that found no effective weight reduction and no significant differences in cholesterol levels, triglyceride levels, and high-density lipoprotein cholesterol levels in schizophrenia patients who had taken ALA (Li et al., 2017; Sanders et al., 2017). However, this observation is in contrast with other studies that have found a reduction in body weight after ALA administration in schizophrenia (N. W. Kim et al., 2016) or obese patients (Huerta et al., 2015; Li et al., 2017). Probably, the reason for the discrepancy among these data can be associated to the different mechanisms responsible for weight gain and dyslipidaemia in patients treated with atypical antipsychotics compared with those not using this class of drugs, or with those who already presented MetS before taking antipsychotics. The metabolic effects of atypical antipsychotics can obviously occur both at the central (hypothalamus and brainstem) and at the peripherical level: neurotransmitters, neuropeptides, and hormones (such as insulin, ghrelin, leptin, and other adipokines) can cause a neuroendocrine imbalance and thus dysregulation of energy control and body weight through increase in appetite and blunting of satiety perception (Canfrán-Duque et al., 2013; Matsui-Sakata et al., 2005). Conversely, in non-antipsychotic-induced MetS, the role of insulin resistance is predominant, together with altered visceral adipose tissue function and adipokines (i.e. leptin, resistin, and visfatin), proinflammatory cytokines (TNF, IL1, IL6), and prothrombotic molecules (PAI-1) production, which strongly influence lipid and glucose metabolism (Alberti et al., 2005; Rader, 2007). These pathophysiological differences can justify the lack of efficacy of ALA on dyslipidaemia and BMI, in our sample. In addition, our results showed significant reduction in AST, γGT, and CPK parameters thus suggesting a potential efficacy of ALA in the control of liver dysfunction in schizophrenia patients.

In the present study, ALA significantly reduced prolactinaemia after 12 weeks of treatment. Antipsychotic-induced hyperprolactinaemia provokes suppression of the hypothalamic–pituitary–gonadal axis with consequences such as sexual dysfunction, adverse effects on bone mineral density, and osteoporosis (Besnard et al., 2014; O’Keane, 2008). Common choices for the management of hyperprolactinaemia include switching the patient to a prolactin-sparing antipsychotic or additional use of D2 receptor agonist such as bromocriptine or cabergoline, however with increasing risk of adverse effects (Haddad and Wieck, 2004). Our findings suggest that ALA can potentially be used to prevent and/or treat the collateral effects of hyperprolactinaemia, avoiding the risk of switching to another antipsychotic or additional medications.

An unexpected and interesting finding was the significant reduction in QTc interval in patients after ALA administration. No studies explored the relationship between the role of ALA and ECG parameters. Psychiatric drugs such as antipsychotics, seem to result in QT prolongation, and that is why it is considered a significant limiting factor when prescribing medications. (Beach et al., 2018). Our results suggest ALA to be a risk-reducing intervention on QTc interval control. The ALA effects on ECG parameters in psychiatric patients can be the subject of further investigations.

## 5 Conclusions

In conclusion, our findings indicated that adjunctive ALA treatment at the oral dose of 600 ​mg/d administered for 12 weeks resulted in a statistically significant reduction of relevant metabolic parameters. The primary result from this study reflects the benefits of ALA regarding blood glucose and confirms that oral administration of ALA can help improve glycaemic status in schizophrenia patients and encourages its use as an adjuvant therapy in atypical antipsychotics-induced diabetes. The unexpected reductions in QT interval and in hyperprolactinaemia are novel findings to be considered for further study, since these two parameters represent vital issues for psychiatrists in clinical practice. Finally, administration of ALA at a dose of 600 ​mg/d was well tolerated during the 3 months of treatment, given the observed absence of adverse events, ALA appears to also be neutral regarding psychopathological symptoms, suggesting its safe use as an adjunctive in psychiatric therapies.

The findings of the present study need to be interpreted with caution because of the presence of several limitations such as the low dosage used, the small sample size, the lack of a comparative treatment, and the uneven distribution between sexes. Additionally, the length of the study was relatively short and thus it was not possible to assess the long-term effects of ALA supplementation. Furthermore, no safe conclusions can be drawn about patient compliance, as therapeutic drug monitoring has not been performed despite its importance in optimizing a treatment. Further clinical trials with adequately powered and well-designed methodology (randomized controlled clinical trials with long-term follow-up) are required to better evaluate the effectiveness of the ALA on atypical antipsychotic-induced metabolic side effects.

## Author statement

Fiammetta Iannuzzo: Writing – original draft, managed the literature searches and wrote the first drafts of the manuscript. All Authors contributed to and have approved the final manuscript. Gianpaolo Antonio Basile: Writing – original draft, managed the literature searches and wrote the first drafts of the manuscript. All Authors contributed to and have approved the final manuscript. Domenica Campolo: Data curation, Formal analysis, organized recruitment, collected the data and undertook the statistical analysis. All Authors contributed to and have approved the final manuscript. Giovanni Genovese: Data curation, Formal analysis, organized recruitment, collected the data and undertook the statistical analysis. All Authors contributed to and have approved the final manuscript. Gianluca Pandolfo: Data curation, Formal analysis, organized recruitment, collected the data and undertook the statistical analysis. All Authors contributed to and have approved the final manuscript. Loretta Giunta: Supervision, Methodology, Formal analysis, supervised the Method procedures, analysis and interpretation of the results, and revised the manuscript. All Authors contributed to and have approved the final manuscript. Domenica Ruggeri: Supervision, Methodology, Formal analysis, supervised the Method procedures, analysis and interpretation of the results, and revised the manuscript. All Authors contributed to and have approved the final manuscript. Antonio Bruno: Writing – original draft, Formal analysis, designed the study, wrote the protocol, and supervised the various drafts and the final version of the manuscript. All Authors contributed to and have approved the final manuscript. Antonino Di Benedetto: Writing – original draft, Formal analysis, designed the study, wrote the protocol, and supervised the various drafts and the final version of the manuscript. All Authors contributed to and have approved the final manuscript.

## Declaration of competing interest

The authors declare that they have no known competing financial interests or personal relationships that could have appeared to influence the work reported in this paper.
